# ‘In most supermarkets food does not cost £3 per day …’ The impact of the school food voucher scheme during COVID‐19

**DOI:** 10.1002/berj.3828

**Published:** 2022-07-14

**Authors:** Gurpinder Singh Lalli

**Affiliations:** ^1^ Institute of Education University of Wolverhampton Walsall UK

**Keywords:** Capabilities Approach, food insecurity, poverty, school food voucher, school meals

## Abstract

Households with children eligible for Free School Meals are at risk of food insecurity. This paper reports on a rapid‐response study that investigated the impact of the school food voucher scheme during the COVID‐19 crisis on young people, families and schools. It pays close attention to the reliance of the state on the goodwill of society and its citizens in feeding those most in need. The Capabilities Approach is used to highlight factors that inhibited and restricted the use of the vouchers to produce the capability of having good nutrition for children in need of Free School Meals. The approach moves towards creating a society where children and young people are able to lead a life of their own choice and contribute to key policy decisions. This qualitative study funded by the British Education Research Association was conducted between September 2020 and March 2021. The study posed two research questions: (1) how have schools responded to COVID‐19 in relation to food during holiday provision; and (2) what have families identified as barriers to accessing the school food voucher scheme? Data collection involved online interviews with young people, schools and organisations (i.e. public health, director from the food industry. etc.). The findings highlight the difficulties with accessing and using the school food voucher and implications for future policy directions. Owing to this being a small‐scale study, it is not generalisable to the wider population but does highlight localised issues.

**Key insights**

**What is the main issue that the paper addresses?**

The main issue the paper addresses is the impact of the continuous reliance on the good will of society and its citizens to support those in deprivation during difficult periods such as the pandemic.

**What are the main insights that the paper provides?**

The pandemic has exacerbated the continuity of how the state relies upon schools and third‐sector organisations to continue feeding children. The paper provides insights into how schools operated during the pandemic in terms of feeding pupils.

## INTRODUCTION

‘When news broke that schools were closing, my first concern was food – learning came second,’ said Katie Barry, head teacher at St. George's Church of England Community Primary School in Gainsborough, Lincolnshire. ‘It feels awful to say that, but we knew not having food would be the biggest issue for families.’ (Human Rights Watch, 2020)

The right to food is enshrined in international human rights treaties, signed by the UK, and measures to ensure children have access to adequate, nutritious food are set out in Article 27 of the United Nations (UN) Convention on the Rights of the Child (UNICEF, 2018). Food is not simply a commodity or biological product, it is also a matter of identity and social value (Lang et al., 2021). In March 2020, in the early days of the first UK‐wide lockdown, the Department for Education (DfE) implemented a shopping voucher scheme worth £15 per child per week in England to provide support for children who would normally receive Free School Meals (FSM) (Parnham et al., 2020). Free School Meals is a benefits‐driven system which provides children with food in schools based on eligibility criteria, such as income support (DfE, 2018). These can be hot and cold meals served in school. Quite often, food is now being transported to schools as opposed to being prepared and cooked on site. According to the Education and Skills Funding Agency (ESFA), mainstream schools should use their core budget allocations (funded through ESFA) to provide FSM to pupils, so government‐funded (maintained) schools are to use their existing budget as pupils from socio‐economically marginalised backgrounds already attract additional funding (ESFA, 2022). During this time, reflecting on the issue of food insecurity led to key questions being raised. Is food a public law issue as opposed to simply being a public health issue? How should universality of school meals be determined? These are concerns which continue to be raised in school food policy. In the absence of the right to food, what can be done to do more for food security in the UK? These are questions which continue to go unanswered. The COVID‐19 pandemic exposed the vulnerability of the ‘market‐oriented food system which can supply food but not necessarily distribute it fairly or meet particular social or cultural needs’ (Shields, 2020, p. 111). The reliance on the third sector such as food banks (Lambie‐Mumford, 2018) and community food services is often said to cover these needs and the UK government has received criticism around issues of accountability (Shields, 2020). This paper draws from a study titled ‘The free school meal voucher scheme and children's access to food during the COVID‐19 crisis’ (Lalli, 2021) and adopts a sociological lens to highlight the impact of the school food voucher scheme using a Capabilities Approach.

Data gathered by the Food Foundation (2020) in late April 2020 indicated that 5 million people in the UK living in households with children under 18 had experienced food insecurity^1^ since the first lockdown started. Of these, 1.8 million experienced food insecurity owing to food supply problems in shops, which meant that 3.2 million people (or 11% of households) suffered from food insecurity owing to other issues such as loss of income or isolation. The Food Foundation (2020) also discovered that, 1 month into lockdown, the parents of 2 million children said that they had experienced one or more forms of food insecurity, and that more than 200,000 children had skipped meals because their families could not access food during lockdown. Therefore, households with children eligible for FSMs were, after 7 weeks of lockdown, at an elevated risk of food insecurity, as they would typically access food in school. This was documented by the Human Rights Watch (2020). For example:

The government's failure to properly ensure all pupils had sufficient food as soon as it closed schools means children have been going hungry. (Kartik Raj – Western Europe researcher at Human Rights Watch; Human Rights Watch, 2020)

There is evidence that COVID‐19 has dramatically widened inequalities in food security and nutrition (Food Foundation, 2021). In the month following the first UK lockdown (from 16 March 2020), 49% of eligible children did not receive any form of FSM, which demonstrates that the voucher scheme did not serve children and young people adequately (Parnham et al., 2020). One study conducted by Parnham et al. (2020) in the UK to investigate access to FSMs among eligible children described factors associated with uptake and whether receiving FSMs was associated with measures of food insecurity using the COVID‐19 wave of the UK Household Longitudinal Study. A questionnaire with 635 children who were eligible for FSMs was included in the data (Parnham et al., 2020). Among FSM‐eligible children, the lowest‐income children were more likely to access FSMs. Receiving a FSM was associated with recently accessing a food bank. On the whole, the study highlighted that the voucher scheme did not adequately serve children who could not attend school during lockdown. Therefore, the study concluded that the FSM vouchers were not an acceptable substitute for standard FSM provision (Parnham et al., 2020).

To draw on an international context, school meals are often taken for granted as it cannot be assumed that all pupils eat the whole school meal everyday (Pellikka et al., 2019). Over half of pupils who would have received FSM at school in England stated that, across a 3‐day period around 2 months after the initial closure of schools, they had eaten no fresh vegetables (Defeyter & Mann, 2020). In the absence of statutory provision, local authorities and third‐sector organisations implemented delivering food through holiday clubs in socio‐economically marginalised communities (Defeyter et al., 2020; Mann et al., 2020). Many UK families with school‐age children face ‘holiday hunger’ owing to the lack of adequate levels of nutritious food available during school holidays (Sretesky et al., 2020). In response to the issues with the scheme raised by many parents and schools, the devolved governments in Scotland, Wales and Northern Ireland chose to introduce alternative schemes (Defeyter et al., 2020). These concerns also prompted an investigation into how the DfE set up and implemented the FSM voucher scheme, and how much it cost, by the National Audit Office, which published its report on 24 December 2020 (NAO, 2020). Despite the problematic rollout of the school food voucher scheme, on 13 January 2021 – amid the third national lockdown – the UK government announced that it would relaunch the scheme using the same provider, Edenred Group. This paper presents a discussion on the difficulties with accessing the food voucher scheme reported by both parents and schools in England. Households with children eligible for FSM are at risk of food insecurity, and for this reason it is critical to shed light on the impact of food on young people and holiday provision, with a view to developing a set of recommendations in finding ways of learning to lead during times of crises.

### Free school meal eligibility across the devolved nations

Firstly, since the beginning of the COVID‐19 pandemic, the number of children eligible for FSMs rose in England and Wales (BBC News, 2021a). Eligibility varies slightly between England, Wales, Scotland and Northern Ireland owing to nations setting their own rules. For example, FSMs during term‐time have been partially funded by the government for more than a century and any new claims made in England must include households earning a maximum of £7400 a year after tax, not including benefits.^2^ In Wales, eligibility depends on a number of factors but must also consist of households who earn no more than £7400.^3^ In Scotland, families are eligible to apply for FSMs based on a number of factors and also if the family income is lower than £16,105.^4^ School food policy approaches differ across the devolved nations and further research on this topic would help to understand the complexities. During the COVID‐19 pandemic, in Wales, it was reported that more than one in five were eligible for FSMs as a further 19,000 children became eligible (BBC News, 2021b). In the Republic of Ireland (Darmody, 2021), the delivery of school meals offers funding of food services of socio‐economically marginalised school children through two schemes: (1) the Urban School Meals Scheme operated by local authorities and part‐financed by the Department of Social Protection; and (2) the non‐statutory School Meals Local Projects Scheme (Department of Social Protection, 2022). It is clear that the way school meals are organised differs significantly among nations and this is documented in the Polish Eurydice Unit (2016) more closely. For instance, in Finland, school meals as a social role which is reflected in policy. In Slovakia, school canteens are founded by local government authorities and registered in the school network. In Spain, the school canteen is a complementary educational services whose existence and recognition are included in the 1990 Act on General Education and part of school life and planning. In Slovenia, schools are required to determine the content, amount and education and training related to meals to encourage the food culture in the annual working plan (Eurydice Unit, 2016). For this study, the focus is specifically on England, and the DfE commissioned the Edenred Group^5^ to help families gain access to school food vouchers during both term time and throughout the holidays, although initially it was only under protest that the vouchers were extended to cover holiday periods (Lalli, 2021).

Whilst the free school meal voucher (during COVID‐19) has been discussed across disciplines of public health (Parnham et al., 2020) and law (Shields, 2020), coverage using a Capabilities Approach in education has been limited. This paper aims to discuss key findings from an empirically informed descriptive study (Lalli, 2021), applying the Capabilities Approach as a conceptual framework, which is introduced below.

## SCHOOL FOOD [IN]SECURITY: THE CAPABILITIES APPROACH

In order to engage in critical discussions on food insecurity, it is useful to draw on the work of the Capabilities Approach as the theoretical framework, developed by Sen (1999). Martha Nussbaum's development of the Capabilities Approach based on two clusters that she identifies is introduced here, the first focusing on ‘quality of life’ and the other on theorising about justice (Nussbaum, 2011). Furthermore, a contextual framework introduces key terms including *wellbeing*, *community* and *agency*. The focus for this study is on the lived realities of schools during the pandemic, their pupils and families who suffer with a lack of access to food in England. This paper highlights the impact of the school food voucher scheme, which was designed for those children eligible for FSM during the COVID‐19 pandemic. The conceptual framework involves embracing the concept of capability as discussed in relation to food education by Hart and Page (2020), but includes an additional layer of *wellbeing* in discussing children's opportunities for access to food.

The use of theory to both foreground and create theories of change is important when interpreting the content of this paper. Freedom is integral to Sen's (1999) approach, which distinguishes between people's capabilities (i.e. what they can potentially be and do) and what they choose to do with them (i.e. how they function). For Sen (1999), capability is defined as a person's real freedom to live in a way they have reason to value. Wider social, economic, cultural and environmental factors have an impact on wellbeing and can provide both carriers (someone who conveys) and enablers (someone who is able to enforce). Food security is a substantial problem in nearly every advanced capitalist nation and is viewed in the light of these carriers and enablers as opposed to simply being a personal matter of working towards a balanced diet driven by calorific intake (Long et al., 2020). The Capabilities Approach offers an account of ‘wellbeing and any account of capability will require some account of wellbeing’ (Robeyns, 2017, p. 118). In developing the theoretical frame for this paper, the Capabilities Approach is the framework for ‘conceptualising wellbeing for public policy that defines wellbeing in terms of what an individual can “do” and “be” in their life’ (Al‐Janabi et al., 2012, p. 168).

When thinking about the Capabilities Approach, it is important that individuals should decide on their own valued freedoms and functionings. When thinking about Nussbaum's Capabilities Approach, this paper dwells on the purpose of schooling in light of universal human flourishing, which should be to provide children with the ability to lead a life of choice and food as a right, but more specifically, school meals are an important social good which allows children to make choices in other social domains/areas of their lives (Earl & Lalli, 2020). Families who have become food insecure may differ along a spectrum based on different levels of poverty, which might include absolute or relative poverty, although such measures and labels become problematic as they are based on social, economic, political and cultural assumptions and contexts, which need unpacking. Also important to recognise is the role of the Capabilities Approach as offering a contextual approach, which places responsibilities and duties on communities and services (Stickley & Wright, 2011). Therefore, this paper moves forward with the lens that Sen (1999) adopted to wellbeing, which is to focus on the local functions that make up a ‘good life’. This paper draws on a model of the Capabilities Approach presented by Goerne (2010, p. 7), who developed a diagram to illustrate the building blocks of the Capabilities Approach (see Appendix 1).

This paper notes the school food voucher as the resource, and the conversion factors are the issues of accessing and using the voucher, which then impact upon an individual's capability set. The functionings are related to what individuals do and gives recognition to the diversity of individuals as they do not all require the same amount or quality of commodities to achieve the same functionings (Goerne, 2010). This paper takes forward the definition of *capability wellbeing* based on the work of Al‐Janabi et al. (2012), who focus on basic capabilities such as being able to be nourished, and paying close attention to such capabilities can be useful for public policy in terms of universalising (Nussbaum, 2011) entitlement to meals.

As an ideological concept, the term *community* lends itself to the sociological imagination and providing an exploration of the meaning of community supports the overall argument which is foregrounded in this paper (Watson & Bogotch, 2016). This paper notes how community varies by context and purpose, but in relation to schools, the term is used in two distinct ways. Firstly the school itself is a community and secondly, the school's engagement with its surrounding neighbourhood is deemed a practice of community (Watson & Bogotch, 2016, p. 94). The notion of community within this context evokes feelings of trust, safety, love and fellowship, but community can also be identified as irreparable when its members are engaged in continuous cycles of poverty (Limperopulos, 2014). Paying close attention to the meaning of community is crucial in theorising the findings in this paper.

To take the concept of agency, it is useful to refer to the work of Gombert et al. (2017), who discuss the conversion factors, as ‘someone's capability to be healthy is a function of his or her access to resources’, such as accessing the school food voucher, ‘and various psycho‐social capabilities that allow him or her to translate those resources into healthy behaviour and ultimately health’ (p. 147). Sen places an emphasis on how the translation of resources into capabilities is subject to variation (Lewis, 2012), based on an individual's characteristics and circumstances (socio‐economic background, gender, class, race, disability etc.). Therefore, the potential for internal (i.e. a lack of access to transport for families trying to use vouchers) and external (limitations in range of supermarkets in which to use the vouchers) barriers to converting resources into actual opportunities (capabilities) and ‘good living’ is recognised, along with the ways in which capabilities interrelate, or convert into one another. For example, the nourishing of socio‐economically marginalised children to be able to engage in learning about making good food choices but lack access to nutritious food, ‘can be said to restrict their agency due to discrimination or disadvantage’ (Lewis, 2012: 5). Also important to note is the potential co‐existence of such socio‐economical marginalisation, for instance, being labelled as a ‘free school meals child’, through which stigma emerges, and this has an impact on the conversion of resources such as FSMs into the capability to be well nourished, and also the conversion of this capability into a wider capability set/capability wellbeing for ‘good living’ (Sen, 2010: 258).

## METHODS AND DATA

One of the problems of conducting research on school food is the variation of systems and approaches across the devolved nations. Whilst the dataset is derived from across the UK and Republic of Ireland, the issues of food insecurity are widespread, but the paper is better interpreted with links to England given that this is where the largest proportion of participants resided (*n* = 12), which includes teachers, parents and pupils. Interpretivism focuses on smaller numbers with an in‐depth analysis of human behaviour (Basit, 2010). Interpretivism is based on the idea that, as individuals, social reality is constructed on an individual basis, through the mind and that ‘only we individually are able to experience the world through personal perceptions, which are manifested through our preconceptions and beliefs’ (Nudzor, 2009, p. 125). This statement is particularly relevant as it supports the methodological rationale for selection. This project involved a small sample of 20 online interviews with participants across the UK (all given pseudonyms), and one from the Republic of Ireland, between September 2020 and November 2020. This is not a representative sample owing to the focus largely being on 12 of the 20 interviews and based in England. However, these interviews form part of an iterative process as the data will be used for follow‐up work on school food policy research across the UK. They included five school catering managers, four school leaders (two headteachers and two assistant headteachers), three young people aged 14–16, one lunchtime supervisor, one public health nutritionist, one food writer and doctoral student, one director of a food organisation, one health visitor, one poverty and inequality commissioner, one health promotion educator and one school food coordinator.

An opportunity sampling approach was used to collect the data, based on the lack of availability during the COVID‐19 pandemic, which meant that participants would often withdraw at short notice from research. The rationale for selecting the above sample involved contacting colleagues within my network who could introduce me to relevant participants across the devolved nations. The rationale included extending beyond networks and placing a call through social media for participants. Therefore, the knowledge and attributes of myself as the researcher allowed me to explore local knowledge and identify samples (Jupp, 2006). The interviews were conducted with participants who reside and work in all four UK nations – England (*n* = 12), Scotland (*n* = 3), Wales (*n* = 2) and Northern Ireland (*n* = 2) – and one participant from the Republic of Ireland (*n* = 1). The 20 online interviews involved three sets of questions aimed at the school leadership, policy and young people (see Appendix 2), with each interview lasting between 20 and 40 min. The interview questions were devised in collaboration with participants and remained open. Data were analysed using thematic and descriptive analysis in order to draw out key themes to respond to the research questions. The qualitative data analysis involved three distinct processes: (1) noticing things, which involved paying attention to the transcripts and following up with participants in case of incomplete responses; (2) collecting and sorting, gathering open codes to learn more about emergent themes from the data; and (3) thinking about things, developing a narrative on each theme and using the data to drive the conversation forward (Miles & Huberman, 1994). For this research, the framework developed by Boyatzis (1998), which offers rigour in producing reliable data for analysis, was adopted to code the data from the interviews. Following this coding process, the framework developed by Braun and Clarke (2006) to thematically analyse the data was used. The limitations to this research stem from the relatively small sample of participants and whilst data saturation occurred, the extent of generalisability is limited (Cohen et al., 2007). However, nonetheless the study draws on experiences during the COVID‐19 pandemic. The limitations also include the short duration of interviews, which in some cases was 20 min, but the conversations before and after interviews meant that rapport could be built, particularly with participants unknown to me. The study was conducted in line with BERA's (2018) Ethical Guidelines for Educational Research.

## FINDINGS

Key findings from this study have been organised into two sections, reflecting key themes and include: (1) school as communities – learning to lead; and (2) barriers for families – reliance on good citizenship. The first section highlights the complexity of thinking about schools as communities, which was presented as part of the theoretical frame for this chapter. The second section reflects the problems vulnerable families experienced with gaining access to food and through the Capabilities Approach (Sen, 1999), in order to problematise the influence of conversion factors (see Appendix 1), which might prevent a focus on the quality of life. Often, achieving this involves removing obstacles in people's lives so that there is more freedom to live the kind of life which individuals find valuable (Gombert et al., 2017). The notion set out by Sen (1999) is based on individual agency, which is crucial in order for individuals to decide for themselves on a valuable life. It is useful to apply the Capabilities Approach as the theoretical framework whilst drawing on the context framework giving reference to wellbeing, community and agency. Furthermore, data from participant interviews thematically account for the impact of the school food voucher on the lived realities of schools, their pupils and families and make meanings using the Capabilities Approach.

### Schools as communities: Learning to lead

The views and experiences from schools are presented here, following conversations with head teachers, and below are responses across primary and secondary schools on how they responded during the first lockdown in March 2020. Schools remained open, one of which acted as a hub school^6^ to take in other children from different schools which were unable to accommodate pupils owing to staffing shortages. Therefore, food and feeding were identified as a focal point when giving consideration to the impact of school closures and it is particularly important to note that all schools simply did not close. For example,

What was happening was that many other local schools just didn't have the staffing capacity to stay open and with us being one of the largest schools the local authority spoke to us about whether we could become a hub school and whether we remained open and took children from other schools. Over the Easter break we had children from 10 different schools with us.

… when the government said they wanted schools to make an offering to key workers and to vulnerable children – we wanted to make sure that we could do that in the best way possible and we stayed open. We didn't close for a single day throughout lockdown.

It quickly became apparent that food was a huge issue. We started to think, okay, what can we do? Obviously the voucher system was there, but wasn't working properly, and that's what led us to start the food distribution programme. (Head Teacher 1)

The data illustrate the impact of conversion factors in families being able to access food safely. For example, some families who did not have access to transport could not travel to supermarkets. Additionally, the voucher system not operating correctly meant that some schools physically delivered food parcels to families. Another head teacher spoke about the lived realities of some families in terms of their background and lack of access to food. Very little is known in the UK about how economic and policy changes impact on families and their ability to feed themselves (O'Connell et al., 2018). The head teacher discussed the impact on families whose parents worked long hours. For example,

We kept up with our caterers, we kept up hot meals, because one of the vital things about that is making sure these children had a hot meal. For those, obviously, from backgrounds where they're impoverished, so it's keeping that up, but also, those from crucial services, it was making sure that they also had a meal, because obviously, their parents would have been working long hours. (Head Teacher 2)

One Assistant Head Teacher discussed how he had travelled across the country on a charity bike ride in order to fundraise as funding was very limited for children and their families. There have been growing concerns about the influence of business and business values in education and little attention has been given to ways in which schools become increasingly engaged in the ‘business’ of fundraising for charities (Power & Taylor, 2018), and in this case funding for families in need of food. The data presented below reflects the involvement of an Assistant Headteacher in feeding families. For example,

I've delivered free school meals to children during COVID, during the lockdown, and then after that had finished, after 17 weeks I then went on a bike ride for children struggling with holiday hunger.

… the charity Meals & More, my target was £5,000 and I'm not far off £11,000 at the moment. Unbelievably overwhelmed by the support I got on my ride and the money is just – wow. It's going to do so much good for children who are those kids that are struggling, that don't get anything.

during the holidays when they're most invisible [pupils], when we don't see them at school, and we can't give them breakfast clubs and what's happening to those kids at that point. (Assistant Headteacher)

During the pandemic, some schools acted as community hubs, supporting families alongside the National Health Service, which goes beyond the school gates and represents the moral purpose that can be found in the practices of school leaders (Sergiovanni, 1996). To (re)imagine school as community is to identify with the communal process involving members of the school community embracing good practice (Watson & Bogotch, 2016) that is reflective of current realities, and ensure that the lived experiences of key stakeholders are considered when thinking about contingency plans in future.

### Barriers for families: Reliance on good citizenship

When engaging with participants, questions relating to barriers were asked and it was interesting to learn about the extent to which such barriers prevented children from accessing food. Following three periods of lockdown,^7^ much has come to the surface about the government's lack of response to ensuring children are able to access nutritious food. One of those examples is the campaign led by the English footballer, Marcus Rashford, who led a petition which later saw the government reverse their decision on extending FSM to children from low‐income families during the holiday period in England (Guardian, 2020). Drawing on the work of Sen (1985), the Capabilities Approach views poverty as a deprivation of capabilities and in this case an individual or family can be deprived of their capabilities, which includes a lack of financial resources, which relates to the theoretical model by Goerne (2010) (see Appendix 1). Converting these offerings (i.e. school meal vouchers) into capabilities is also problematic as they are not necessarily guaranteed to reach families. The relationship between food intake and nutritional value is analysed through the work of Sen, who discusses the underlying problems of agency and choice which are relevant in the context of food insecurity of vulnerable individuals (Gombert et al., 2017). Below, an account of responses from participants is presented, specifically in relation to such barriers, which include ICT literacy, ‘on‐school barriers’ (social cultural) and a lack of understanding of using the voucher system. For example,

… I think we've got a lot of parents who aren't totally IT literate, a lot of parents who – we are constantly on to our parents about ensuring we've got the correct contact details.

There was the on‐school barrier, which was the fact that we couldn't get on the system. The system kept crashing. Our business manager would be logging on at two in the morning. I think there was a chart showing when the least traffic was going through the site so he'd set his alarm and get up at half past one, two o'clock and start trying to get onto the site.The barriers for the parents which were around the lack of understanding around the voucher system, the fact that when parents went on, often they couldn't access the programme. Also, we had a lot of parents who should have been entitled to free school meals but had never registered. (Head Teacher 1)

The Assistant Headteacher highlighted how parents were unaware of the introduction of this voucher scheme as communication was poor, also noting that whilst the scheme had started, this took 2–3 weeks, so this particular school decided to opt for delivering food parcels so that they could visit the pupils and families. Owing to the lack of information and communication issues, these were contributing factors affecting the ability to turn the vouchers as a commodity into capabilities (Goerne, 2010). These include, being well fed, having good nutrition which relates to capabilities and wellbeing. It was also noted that a reason for delivering vouchers meant less risk to families from leaving the house. For example,

Parents didn't know this was going to happen. They weren't prepared for it, and as one of the families said later on the deliveries, normally during up to the summer holidays she saves money to be able to feed her children through the summer holidays but actually because the kids are home more.

Yeah, that [Edenred Group voucher scheme]didn't come in until – I mean we started on Monday and that didn't come in for probably two or three weeks after I started delivering the meals. We didn't really go for that. There are a couple of reasons we didn't go for vouchers. Firstly, what we were doing was working brilliantly and I was able to see the parents and children every day and make sure things were good. With the voucher scheme that wasn't a possibility. I couldn't see the children.

Then also with the voucher scheme the parents had to leave the house to spend the vouchers which meant leaving the house, putting themselves and their children in danger. Taking three or four kids to the supermarket during COVID restrictions probably wasn't the best idea in the world. It was stressful for everybody. So, although it was a great idea from the government as a backup there were better systems that schools could have done like delivering the meals by hand or some things like that. (Assistant Head Teacher)

So in line with Goerne's (2010) model, the difficulty in gaining access was not merely about eligibility, or the consumption of food, but it was about issues of conversion as access is based on a number of assumptions, for example, based on whether or not families can leave the house to go to supermarkets and whether or not they have the means or transportation for doing so.

Two young people described the impact of the COVID‐19 pandemic on family life and how unemployment affected access to food. From reporting on his father's job loss to having to cover lunch money and the rising tensions of trying to buy nutritious food and make sure the supermarkets would accept the vouchers. It is highlighted that the vouchers did not work for everyone and this illustrates the point about the barriers and conversion factors as discussed in relation to the Capabilities Approach (Goerne, 2010). For example,

They were – of course my dad had just recently just lost his job. So, there was – we had those – like financial turmoil in our family. It was a really scary prospect because of course like you know the mortgage payments had been stopped and everything with that COVID relief.

But the prospect of having to pay for food and having to pay for lunch and this much money that normally I'd be having at school, I'd be having a good, nutritious lunch at school. My parents having to like you know … having to pay for this. It was a scary prospect for them. So, I think about what I know – like yeah, how they'd normally have to compromise on bills etc.

All I know is the schools voucher scheme didn't necessarily work for everybody. They couldn't access vouchers. They'd turn up to the shops and stores to find there's no credit on them and things like that. I don't want to be too negative about it, but a lot of people were saying that it wasn't functioning very well, the way it was set up. (Young person 1)

At my school we got the vouchers, but obviously in most supermarkets food does not cost £3 per day. So, meals were just less consistent. My sister's school for example, they told them that if they wanted to eat they had to go to school to eat. They wouldn't give them vouchers so they would have to go to school for lunch and then come home. Which made no sense and obviously not many kids wanted to commute all the way there and be more exposed and then come back. (Young person 2)

A manager at one of the catering organisations described the importance of a hot meal but also the problems with the voucher scheme itself. This response illustrates the impact of conversion factors at play. For example,

So, the whole voucher scheme, candidly, we weren't a fan of … we would have much preferred the kids to have had food delivered rather than have the option of abusing a system. There's a big difference between a free school meal pupil having a hot meal or even a cold meal in a school, where somebody is aware of safeguarding and pupil wellbeing, then somebody getting something at home where you may or may not be suffering because they're latchkey kids, they're whatever they happen to be in terms of the terrible world that we live in. (Catering organisation)

It is clear that systemic issues in the food system have had an impact on families being able to access food for their children. The lived realities of those involved in supporting families in accessing food during the pandemic are widely cited in the media, during a time which has seen the introduction of a National Food Strategy,^8^ documenting key recommendations for creating a more equitable food system in England.

## DISCUSSION: FOOD INSECURITY AND CAPABILITY

The UK government's agenda on feeding its children has intensified and a recent example of this was evident through a campaign (Guardian, 2020) to increase access to food both during term time and during school holiday periods, throughout the pandemic in England. Drawing on the Capabilities Approach, conversion factors and its impact on wellbeing and community helps to highlight the importance of agency in ensuring that children have access to good and nutritious foods but more importantly that they are able to lead a life of choice. Using Goerne's (2010) agreed upon Capabilities Approach framework, attention was paid to the complex nature of conversion factors and the call for universal capabilities in feeding children and young people in school. After all, society continues to bear witness to those experiencing food insecurity which hinders their capabilities and individual agency of leading a life they value. The data presented in this paper illustrate the stark realities of the impact of conversion factors and reliance on schools for ensuring children have access to food as schools begin operating as food banks in some cases,^9^ particularly during the pandemic. Families who are food insecure experience difficulty in feeding their children or at least feeding them during the holiday period, but as a society, it is important to take joint responsibility and think hard about how to equip families with the agency required to break free from such cycles of deprivation, but doing so through policy reform. However, without properly set‐up voucher systems, families continue to suffer through cycles of deprivation which cannot continue. As a result, children suffer socially and psychologically, which impacts on life chances, and policy decisions tend to rely on the use of FSM as a proxy for measuring socio‐economic marginalisation (Gorard & Siddiqui, 2019; Hobbs & Vignoles, 2010). The reliability of FSM as a proxy for measuring marginalisation has been criticised (Taylor, 2017), as some children who are not in receipt of FSM still suffer from food insecurity (Gorard et al., 2021). More families registered to gain access to the FSM during the COVID‐19 pandemic so consideration to stigmas need to be thought about as these come with labelling (Chambers et al., 2016). At this point, it is helpful to return to the main research questions here by addressing each one:

*How have schools responded to COVID‐19 in relation to the provision of food during holidays?* Based on the findings from this study, the Edenred Group voucher scheme was problematic when it was first launched and, as the data presented in this report highlight, it caused avoidable hardship to young people and their families and created additional difficulties for schools amid a national crisis. The way in which schools responded varied, and it was only possible to capture what schools had done during this period by talking to thems. This demonstrated the need to develop a wider network for school leaders so that schools’ varied activities and approaches can be shared to ensure that schools learn from one another and develop a consensus about what constitutes good practice.

*What have families identified as barriers to accessing the school food voucher scheme*? The barriers identified included a lack of understanding of how to access the voucher scheme and, for some, a lack of ICT skills. They also included the stigma attached to the vouchers, and the fact that they clearly did not provide a budget sufficient for families to feed their children on. This report has highlighted the impact of COVID‐19 on feeding children. However, more importantly, issues around food security already existed prior to the global pandemic and food is understood to be a human right. In this research, providing families with cash funds as opposed to vouchers has been identified as a key recommendation that will ensure that families are able to feed their children immediately during such critical times. In conclusion, context matters, as does school culture, and these need to be considered when developing recommendations for school leaders and policy makers. All of this is documented in the published report (Lalli, 2021) following the completion of the study, which has informed this paper.

### Limitations

Whilst it was difficult to generalise from a small sample, the experiences of young people, policy makers, schools and wider community have been documented and key themes emerged. Having responded to the research questions above, before concluding it is important to highlight the limitations of this research. The study was based on a relatively small sample and interviews were conducted remotely with all participants. This was due to the lockdown, so conducting research face to face was not possible. The work that exists on school food policy was limited so it was difficult to draw on relevant and existing datasets to inform this study, so whilst a limitation, this is also a gap for further research.

## CONCLUSION

In conclusion, it is difficult to draw concrete conclusions on the real impact of the school food voucher scheme and its impact on schools and their communities owing to the small sample size. This paper highlights the continuous reliance on the good will of society and its citizens to support those in deprivation during difficult periods and the pandemic has exacerbated the continuity of how the state relies upon schools and third sector organisations to continue feeding children. Despite the problematic roll‐out of the school food voucher scheme by contractor Edenred Group, the UK government continued to use the same provider in lockdown three on 13 January 2021.^10^ To summarise, food has a huge impact on the daily lives of children, who reported their lack of social interaction owing to school closures. Data revealed that schools responded very differently both locally and nationally. There has been a lack of training for staff and parents on the operations of the scheme. School resources vary in terms of finance, but also in terms of knowledge, specialist skills and capacity.

### Context and school culture matters

Schools are complex in their funding structures as funding models have different implications for different types of schools across nations. Some schools are much better resourced in terms of knowledge and expertise in food. It is important to consider what works in schools in terms of food education. Recognising the importance of food in daily life is crucial in shaping and influencing future citizens. Developing a culture that considers food insecure families in moving school policy debates forward is crucial. Investing in food education matters and it might be useful to consider removing the term ‘free’ from school meals as food is a human right (Lambie‐Mumford, 2018), not a privilege.

### Recommendations for school leaders

It would be useful to have contingency plans for school meals set by leaders in place. In addition, school food needs to be considered as a central part of the community, curriculum and review staff working practices. Schools need to continue engaging with local stakeholders for access to fresh produce and to create seasonal menus. Schools need to continue engaging with local MPs, parent governors and pupil committees. Further engagement with parent governors and pupil committees is crucial in capturing lived experiences moving forward.

### Recommendations for national policy makers

For policy makers, it is important to engage with key stakeholders, i.e. chefs in schools, school leaders, School Food Matters, DfE School Food Units, Food Foundation and Taste Education. In addition, listening to the views of families by holding regular informal focus group meetings is crucial. Furthermore, considering the variability of school structures and deprivation when formulating policy, adopting a more localised approach and moving away from short‐term solutions will help. In terms of school food policy, it is important to advocate for a universal approach to school meals which is translated into policy terms and to learn lessons from other nations.

## CONFLICT OF INTEREST

The author reports no conflict of interest.

## ETHICS STATEMENT

The study was conducted in line with BERA's Ethical Guidelines for Educational Research (2018).

## Data Availability

The data that support the findings of this study are available on request from the corresponding author. The data are not publicly available owing to privacy or ethical restrictions.

## Endnotes

^1^
A household‐level economic and social condition of limited or uncertain access to adequate food. Households are considered food insecure if they experience ‘severe’ or ‘moderate’ food insecurity.

^2^
On eligibility for free school meals in England see https://www.gov.uk/apply‐free‐school‐meals

^3^
On eligibility for free school meals in Wales see https://gov.wales/sites/default/files/publications/2019‐04/free‐school‐meals‐in‐wales‐information‐for‐parents‐and‐guardians.pdf

^4^
On eligibility for free school meals in Scotland see https://www.mygov.scot/school‐meals

^5^
The Edenred Group is a leading digital platform for services and payments, which was commissioned to roll out the school meal vouchers during the COVID‐19 pandemic.

^6^
A school that serves as an example for school improvement to the rest of the state, in terms of excellent for teacher and leadership training and development; https://www.gov.uk/guidance/teaching‐school‐hubs

^7^
For a timeline of UK coronavirus lockdowns, March 2020 to March 2021 see https://www.instituteforgovernment.org.uk/sites/default/files/timeline‐lockdown‐web.pdf

^8^
National Food Strategy: An independent review for government, 2021; https://www.nationalfoodstrategy.org/

^9^
One in five UK schools has set up a food bank in Covid crisis, survey suggests; https://www.theguardian.com/society/2021/mar/04/rise‐in‐food‐banks‐in‐uk‐schools‐highlights‐depth‐of‐covid‐crisis‐survey

^10^
Providing school meals during the coronavirus (COVID‐19) outbreak, Department for Education; https://www.gov.uk/government/publications/covid‐19‐free‐school‐meals‐guidance/covid‐19‐free‐school‐meals‐guidance‐for‐schools

## APPENDIX 1

Five elements of the Capabilities Approach. Retrieved from Goerne (2010).

## APPENDIX 2

### Interview questions

#### Young people

1. Could you describe your experience of lockdown?
2. Did you know about the school food voucher scheme?
3. What did you struggle with most during lockdown?
4. What would you have benefited from during this time?
5. What should the government do to help reduce food poverty?
6. What role could young people like yourself play?

#### School leadership

1. First of all, what is your position in the school and how long have you worked in education?

2. Could you describe your experience of leading a school during a period of national lockdown?

3. What impact has lockdown had on families whose children are enrolled at your school?

4. Were you informed of the school food voucher scheme and how accessible was this to your school?

5. Did you encounter any issues in providing families with information on the school food voucher scheme?

6. Schools are not only places for learning but also for accessing healthy food. Could you describe how children's food intake was affected during COVID‐19?

7. If you had to contribute to developing a toolkit for schools on school food, what would you include?

#### Policy and third sector

1. What is your current position?

2. Could you describe your experience of lockdown in the context of your role?

3. What have families identified as key barriers in accessing the school food voucher scheme?

4. Could you share a story of how schools in your local region have responded to COVID‐19?

5. What have been the notable barriers in families being able to access the School Food Voucher?

6. If you had to contribute to developing a toolkit for schools on school food, what would you include?
